# Right Ventricular Pacing as Rescue Therapy for Cardiogenic Shock in Takotsubo Cardiomyopathy With Left Ventricular Outflow Tract Obstruction

**DOI:** 10.7759/cureus.105198

**Published:** 2026-03-13

**Authors:** Atsuo Mori, Tomoo Nagai, Tatsuji Yoshimoto, Noritaka Shimizu, Yuko Harada, Hirofumi Haida, Tadashi Omoto, Tomoaki Masuda

**Affiliations:** 1 Cardiovascular Surgery, Kawasaki Municipal Hospital, Kawasaki, JPN; 2 Cardiology, Kawasaki Municipal Ida Hospital, Kawasaki, JPN; 3 Internal Medicine, Harada Naika Clinic, Kawasaki, JPN; 4 Cardiovascular Surgery, Keio University, Minato, JPN

**Keywords:** beta blocker therapy, cardiogenic shock, hypertrophic cardiomyopathy (hcm), left ventricular outflow tract obstruction (lvoto), permanent pacemaker implantation (ppm), right ventricular pacing, stress induced cardiomyopathy, systolic anterior movement, takotsubo cardiomyopathy (ttc)

## Abstract

Takotsubo cardiomyopathy (TC) is a disease characterized by chest pain that must be differentiated from acute coronary syndrome, yet the coronary arteries are normal, and left ventricular systolic function is impaired. Historically, it had been recognized as a disease with a relatively favorable prognosis that improved spontaneously, but recent reports have made it clear that this is not necessarily the case. Particularly challenging situations arise when left ventricular outflow tract obstruction (LVOTO) is present, and this tendency is especially pronounced when accompanied by cardiogenic shock. In such cases, beta-agonists and nitrates are contraindicated, and beta-blockers cannot be used due to hypotension, leaving limited treatment options. This report describes the first successful treatment of cardiogenic shock due to TC with LVOTO using right ventricular (RV) pacing. A 79-year-old woman presented with chest pain; coronary angiography was normal. She subsequently developed cardiogenic shock. Echocardiography revealed akinesis from the apex to the septum, hyper-contraction at the base, severe LVOTO with associated systolic anterior movement of the mitral anterior leaflet, leading to mitral regurgitation. Peak pressure gradient (PG) was 160 mmHg in the left ventricular outflow tract, and an ejection fraction (EF) was 30%. Despite fluid therapy and norepinephrine administration, there was little improvement, and systolic blood pressure dropped to 60 mmHg. Upon initiating emergency temporary RV pacing, systolic blood pressure rose from 68 mmHg to 98 mmHg, and the left ventricular outflow tract PG decreased from 160 mmHg to 107 mmHg. Basal hyper-contractility also decreased. On the following day, a permanent dual-chamber pacemaker was implanted to achieve atrioventricular synchrony, leading to further stabilization of hemodynamics. By day 21, cardiac function normalized (EF 60%, PG 7 mmHg). The acetylcholine stress test ruled out coronary vasospastic angina. We diagnosed this patient with TC with LVOTO. The patient was discharged in good condition. Intentional induction of left ventricular desynchrony via RV pacing reduced hyper-contractility, rapidly lessened LVOTO, and facilitated recovery from cardiogenic shock. To the best of our knowledge, this is the first report demonstrating the efficacy of RV pacing in shock patients with TC complicated by LVOTO. In this highly critical situation, it may provide an easily accessible and clinically useful treatment option.

## Introduction

Takotsubo cardiomyopathy (TC) is characterized by transient left ventricular dysfunction, often triggered by emotional or physical stress. While TC was considered to have a favorable prognosis, recent reports have indicated that the in-hospital mortality rate is comparable to that of acute coronary syndrome [1,2]. Particularly in cases with cardiogenic shock, the mortality rate is high at 9.9% [1]. Furthermore, left ventricular outflow tract obstruction (LVOTO) is known to be present in 12.8-25% of cases [1,3]. Cases of TC with LVOTO and shock exhibit significant hemodynamic instability [3].

Beta-agonists are contraindicated as they increase baseline hypercontractility and worsen LVOTO. Beta-blockers are often difficult to administer in hypotensive states [4]. Nitrates reduce preload and may further exacerbate obstruction. While intra-aortic balloon pumps (IABPs) have limitations in reducing high afterload, Impella has been reported to be useful [5-7]. However, its use is currently available only at selected centers. Therefore, a safe, rapid, and effective treatment strategy is urgently needed for these high-risk TC patients.

Right ventricular (RV) pacing theoretically holds the potential to intentionally induce left ventricular desynchrony, reducing baseline hypercontractility, and alleviating LVOTO. However, to date, no successful cases of using RV pacing for cardiogenic shock in TC with LVOTO have been reported. To the best of our knowledge, this is the first report of a patient presenting with shock symptoms of TC with LVOTO who was successfully resuscitated by emergency RV pacing.

## Case presentation

The patient was a 79-year-old female who was an outpatient at the cardiology clinic for episodes of tachycardia due to paroxysmal atrial fibrillation. She exhibited no prior medical history of congestive heart failure or cardiogenic shock. The patient had never undergone cardiac catheterization. The patient's medical history did not include any surgical procedures. The patient's family history included a mother with an unspecified cardiac condition. Prior to admission, there had been no occurrence of psychological distress.

**Figure 1:**
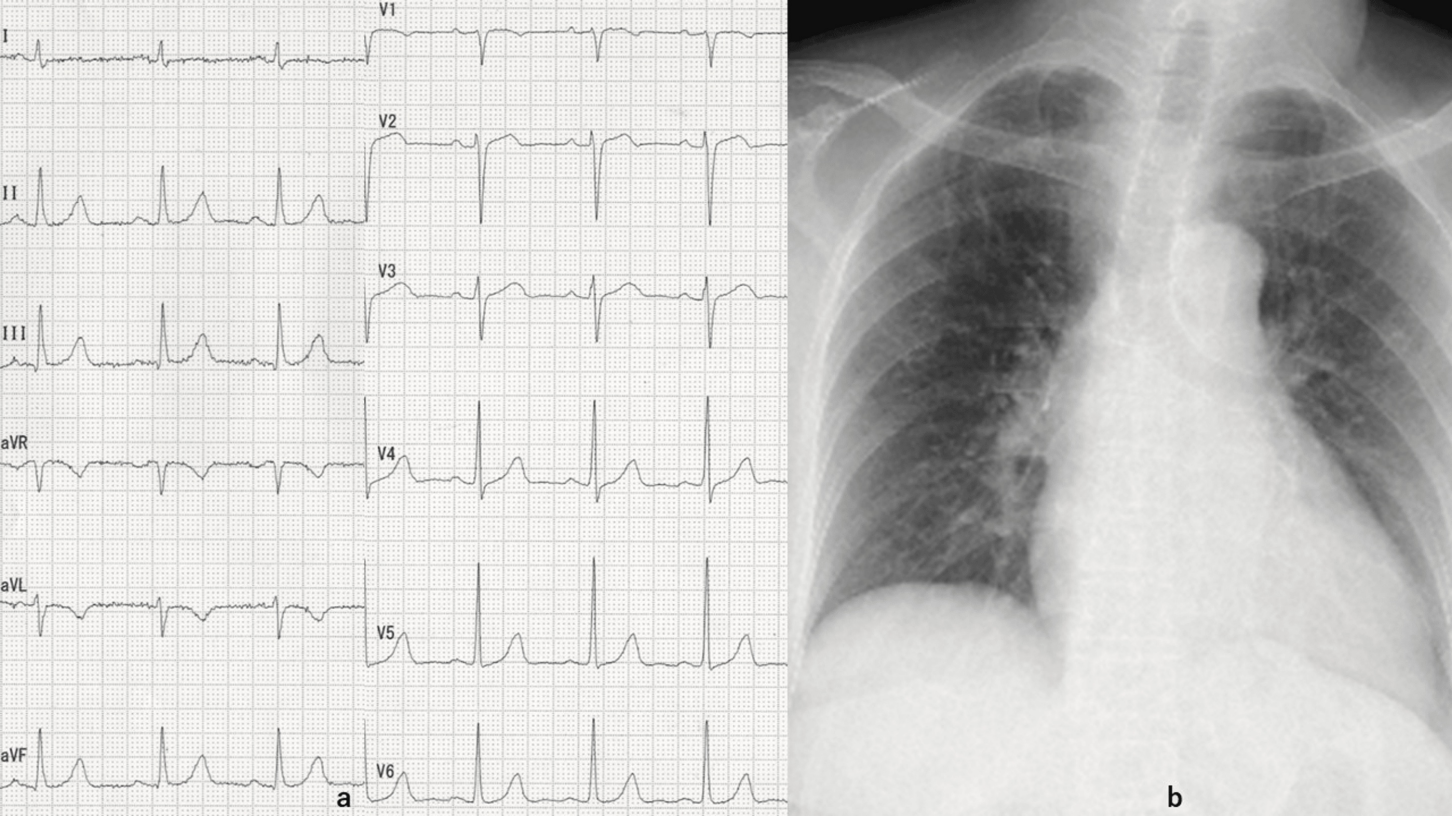
ECG and chest X-ray film at admission a: The resting electrocardiogram (ECG) showed sinus rhythm with a heart rate of 50 beats per minute and no ST-T changes in the chest leads. b: Chest X-ray film showed moderate cardiac enlargement but no signs of pulmonary congestion.

On 18/08/2025, the patient was transported by ambulance to our emergency department with the chief complaint of chest discomfort at rest, which had begun the previous day. Upon arrival, she exhibited vital signs that were within normal limits, with a blood pressure reading of 108/60 mmHg, a pulse rate of 68 beats per minute, and no evidence of arrhythmia. Auscultation revealed no systolic murmur. A chest X-ray (CXR) was performed, which revealed moderate cardiac enlargement but did not demonstrate any signs of pulmonary congestion (Figure 1b). The patient's electrocardiogram (ECG) revealed a sinus rhythm with a heart rate of 50 beats per minute and no ST-T changes in the chest leads (Figure 1a).

The troponin I level was elevated at 394 pg/mL. The B-type natriuretic peptide (BNP) demonstrated a mild elevation. Bedside echocardiography (EC) performed in the emergency room revealed normal ventricular wall motion with left ventricular outflow tract (LVOT) septal bulging. The LVOT pressure gradient (PG) was 10 mmHg. The patient was admitted to the hospital with a suspected case of unstable angina.

**Figure 2:**
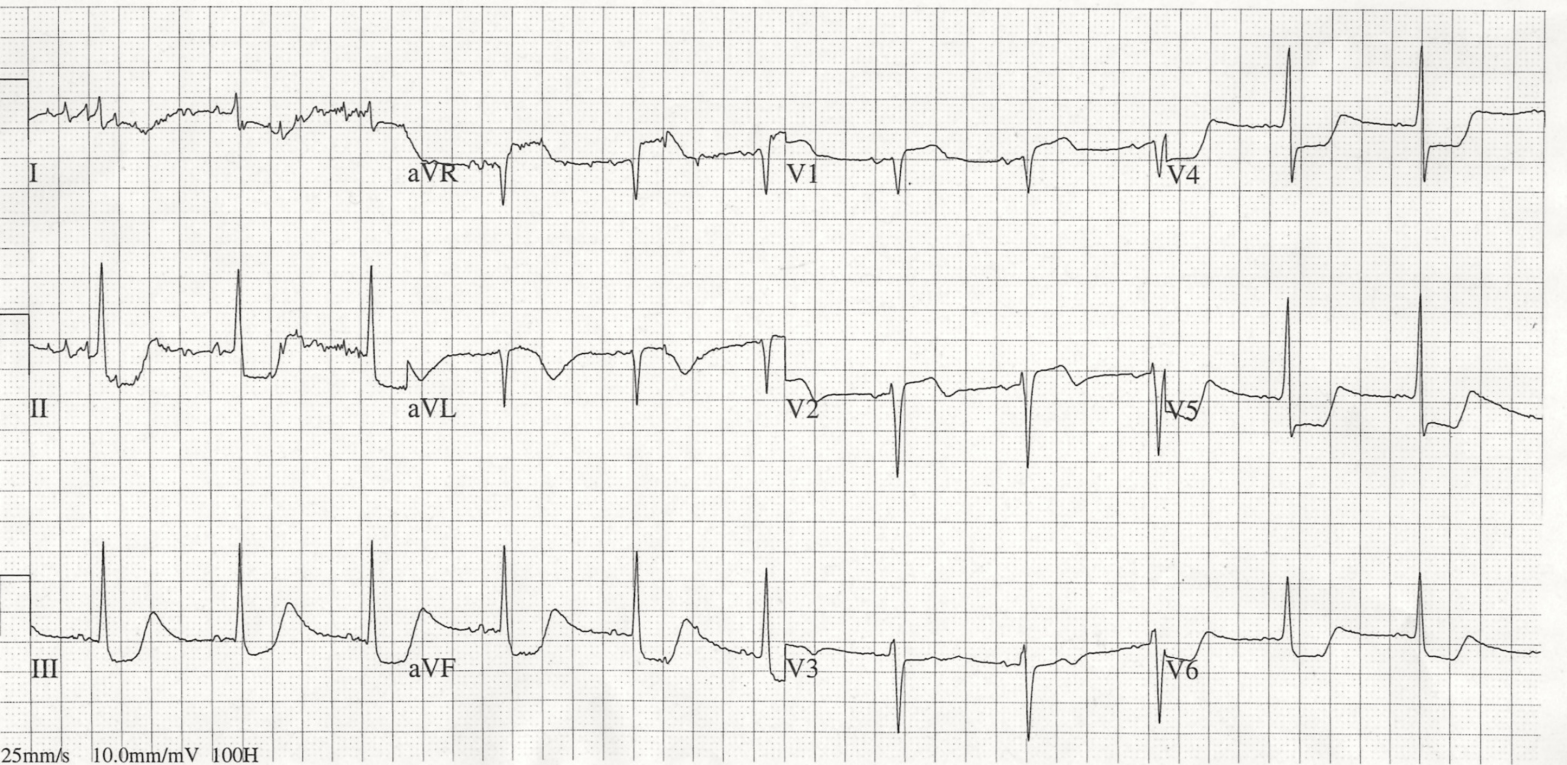
ECG at the time of attack of severe chest oppression A portable ECG showed ST elevation in aVR and V1-V2, and marked ST depression in leads II, III, aVF, and V4-6.

Following admission on 19/08/2025, at 3:00 AM, the patient reported experiencing chest pain. The administration of nitroglycerin resulted in a significant decrease in blood pressure. Concurrently, the heart rate decelerated to 40 beats per minute. The administration of noradrenaline via a peripheral intravenous line resulted in an increase in blood pressure. The patient's chest pain resolved temporarily, but at 7:00 AM, the patient complained of persistent, severe chest pain. A portable ECG revealed ST elevation in leads aVR and V1-V2, as well as marked ST depression in leads II, III, aVF, and V4-6 (Figure 2).

A bedside EC revealed reduced wall motion from the anterior left ventricular wall to the apex. The administration of fluid loading and continuous peripheral infusion of norepinephrine resulted in an increase in blood pressure.

The patient's chest pain temporarily subsided. However, given the suspicion of unstable angina, an emergency coronary angiography (CAG) was performed on the morning of 19/08/2025.

The left coronary artery was normal (Video 1). The right coronary artery showed an abnormal origin but no stenotic lesions (Video 2).

**Video 1:** Coronary angiography showing that the left coronary artery was normal.

**Video 2:** Right coronary artery showing an abnormal origin but no stenotic lesions.

Left ventricular angiography was not performed. In light of the aforementioned findings, it was deemed probable that subsequent to the completion of the CAG, the likelihood of vasospastic angina would be significant. The patient was subsequently transferred back to the intensive care unit (ICU) for further treatment.

Following readmission to the ICU, the patient once again complained of acute chest pain and exhibited a precipitous drop in blood pressure. The systolic blood pressure measured by the sphygmomanometer was recorded at 60 mmHg, indicating a state of severe shock. The ECG revealed ST elevation in leads aVR and V1-2, as well as ST depression in leads II, III, aVF, and V4-6. The administration of norepinephrine via a peripheral intravenous line was initiated at a rapid rate. However, this intervention did not result in the anticipated improvement. Consequently, there was a deterioration in the patient's level of consciousness.

During the bedside EC in the ICU, the left ventricle exhibited ballooning from the apex to the interventricular septum and posterior wall. However, the base was the only component that demonstrated excessive contraction.

**Figure 3:**
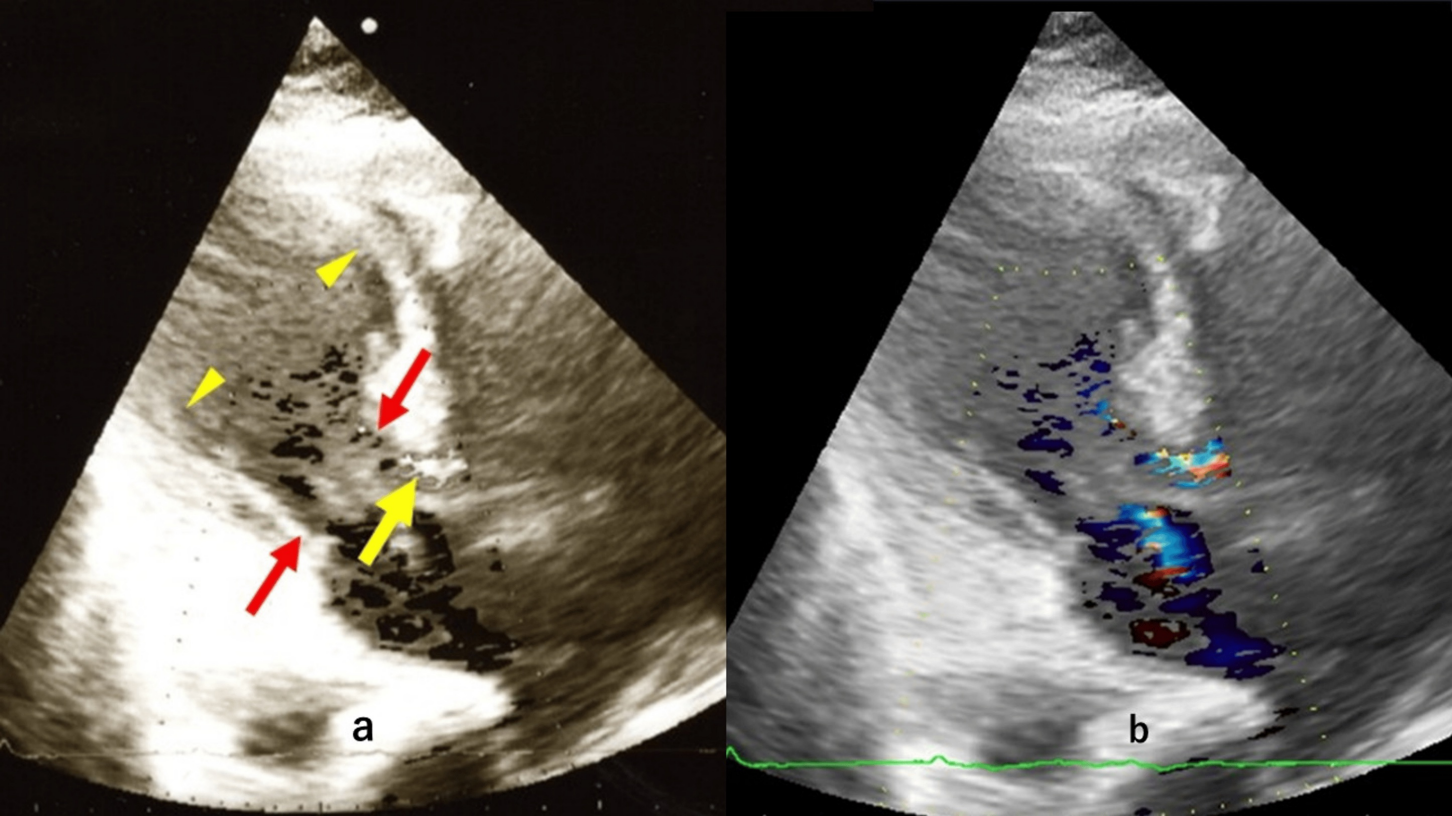
EC in ICU after CAG a: During the bedside echocardiography (EC) in the intensive care unit (ICU) at this time, the left ventricle showed akinesis from the apex to the interventricular septum and posterior wall (yellow wedges), with only the base exhibiting excessive contraction (red arrows). Echocardiographic Doppler measurement revealed a pressure gradient reaching 160 mmHg (yellow arrow). b: Furthermore, there was evidence of the anterior movement of the mitral valve anterior leaflet during systole, and severe mitral regurgitation was also present. CAG: coronary angiography

The EF was 30%. The presence of severe LVOTO was attributed to the occurrence of ventricular septal protrusion into the LVOT, in conjunction with systolic anterior movement (SAM) of the mitral anterior leaflet. Echocardiographic Doppler measurement revealed a PG reaching 160 mmHg. Additionally, the presence of SAM of the mitral valve anterior leaflet during systole was observed, along with severe mitral regurgitation (Figure 3). At this juncture, it was determined that the patient's cardiac condition was consistent with TC. The patient's critical condition, precipitated by shock resulting from LVOTO, was also recognized.

**Figure 4:**
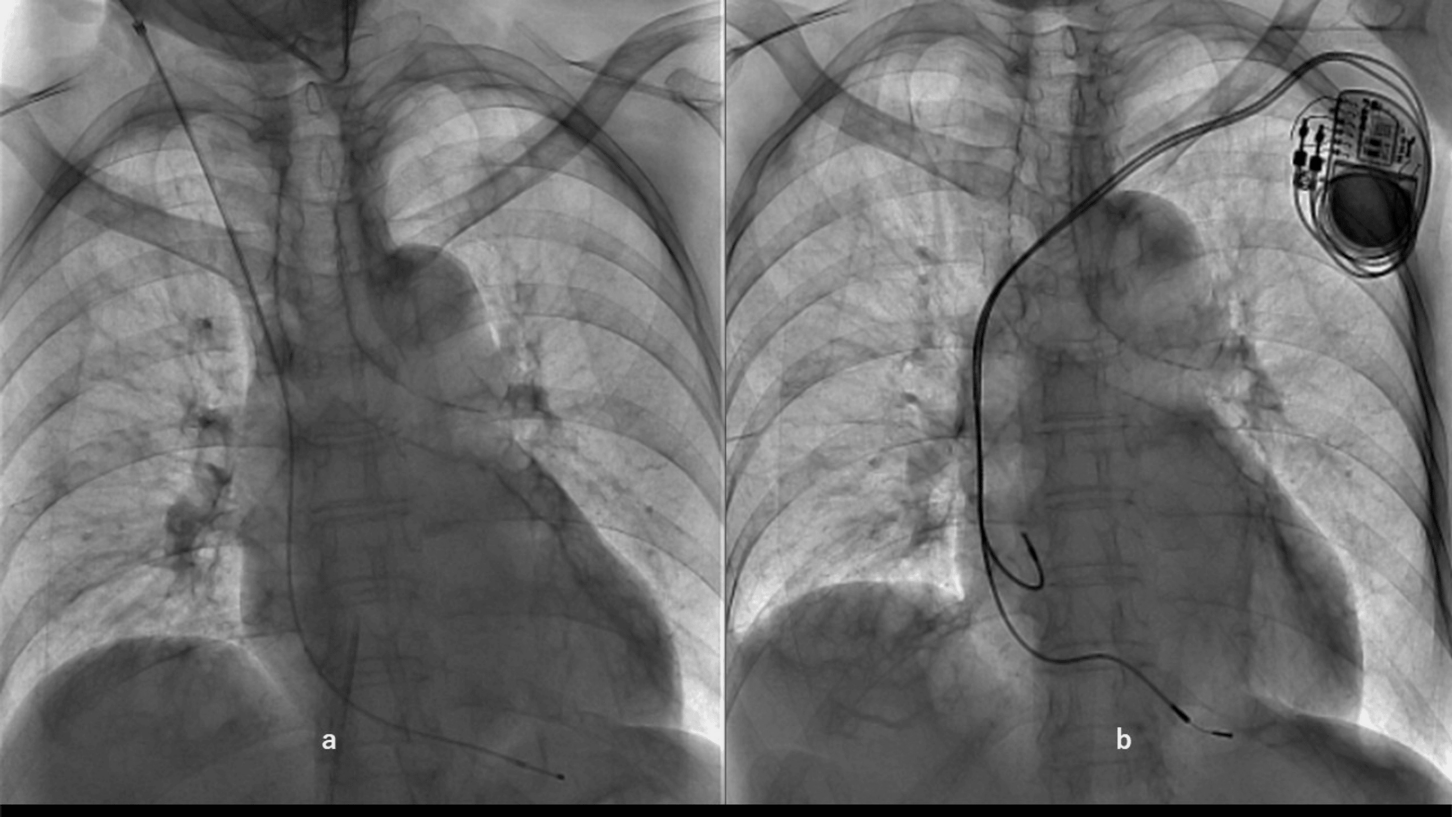
Temporary and permanent right ventricular pacing placement a: Considering the patient's history of prior bradycardia episodes and expecting the improvement of left ventricular outflow obstruction, we placed a temporary external pacing lead from the right jugular vein into the right ventricle. b: A permanent dual-chamber pacemaker was implanted the day after cardiogenic shock developed. The temporary external pacing lead was removed.

Subsequently, we proceeded to the cardiac catheterization laboratory, where we initiated the procedure by securing a central venous (CV) line and an arterial pressure line. The arterial pressure was measured immediately after the arterial line was inserted into the left femoral artery, yielding a reading of 68/31 mmHg, which is indicative of shock. The infusion of rapid fluid was initiated through the CV line, which was inserted into the inferior vena cava via the left femoral vein. The administration of norepinephrine was initiated through the side port. In light of the patient's medical history, which includes bradycardia-tachycardia syndrome and documented instances of bradycardia, a temporary external pacing lead was implanted from the right jugular vein into the right ventricle (Figure 4a).

The rationale was multifaceted: it was considered that beta-blockers might induce severe bradycardia and be difficult to use without external pacing backup. Furthermore, given the established benefits of RV pacing in reducing PG in cases of LVOTO associated with hypertrophic cardiomyopathy (HCM), it was hypothesized that this approach may also yield advantageous outcomes in these circumstances.

**Figure 5:**
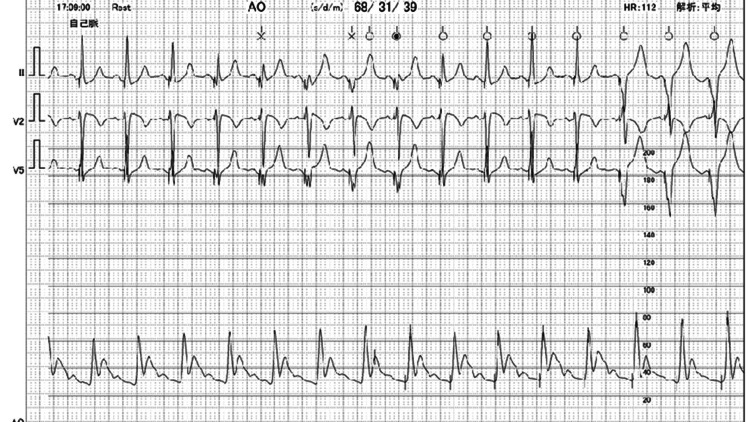
Arterial pressure before temporary RV pacing This shows the arterial line waveform from spontaneous circulation immediately before initiating temporary right ventricular (RV) pacing due to cardiogenic shock. Blood pressure was extremely low at 68/31 mmHg, and the waveform was narrow.

Upon the initiation of temporary RV pacing, the blood pressure measured on the arterial line during pacing was higher than that during spontaneous beats. The spontaneous systolic blood pressure was documented as 68/31 mmHg (Figure 5).

**Figure 6:**
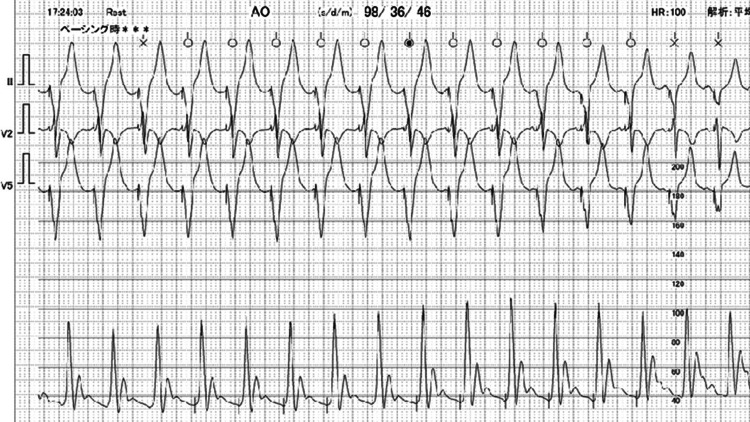
Arterial blood pressure after insertion of temporary RV pacing lead During cardiogenic shock due to takotsubo cardiomyopathy with left ventricle outflow tract obstruction, temporary right ventricular (RV) pacing was initiated. Blood pressure rose to 98/36, but the waveform remained narrow, suggesting low stroke volume.

RV pacing increased the systolic arterial pressure up to 98/36 mmHg (Figure 6).

**Figure 7:**
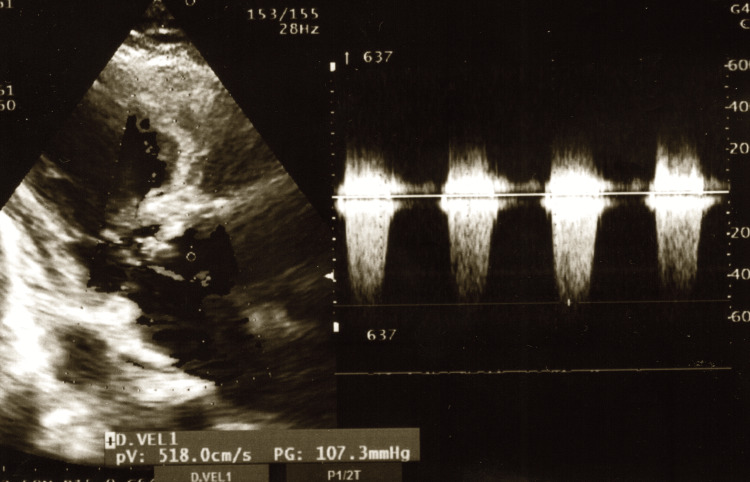
Echocardiography after installation of RV pacing This shows portable echocardiographic findings immediately after initiating temporary right ventricular (RV) pacing during cardiogenic shock. Color Doppler measurement revealed a left ventricular outflow tract pressure gradient of 107 mmHg, a marked improvement from 160 mmHg.

Immediate bedside EC revealed that the PG across the LVOT was 160 mmHg before pacing, but after RV pacing was initiated, the PG decreased to 107 mmHg (Figure 7). These findings suggested that RV pacing resulted in an improvement in the circulatory status.

Subsequent to confirming that the arterial pressure was higher than the spontaneous pulse pressure, the pacing rate was set relatively high at 90 beats per minute to achieve full pacing. There was a gradual stabilization of the patient's blood pressure, and their level of consciousness improved to the point of responding to verbal stimuli.

Several reports have shown that RV pacing reduces basal left ventricular hyperkinesis more effectively than spontaneous rhythm in patients with HCM [3]. These findings support the usefulness of RV pacing, namely, that intentional desynchrony at the ventricular level may reduce LVOTO in TC.

The findings of MR due to SAM remained consistent. At this juncture, the performance of left ventricular angiography to confirm LVOTO was recommended by several experts. However, given the risks associated with persistent hemodynamic instability, the procedure was not performed. In light of the aforementioned findings, it was determined that the patient exhibited characteristics indicative of either TC with LVOTO, coronary vasospastic angina, or a combination of both.

Following the patient's readmission to the ICU, four units of stored blood and two units of fresh frozen plasma were administered to address anemia and augment preload. The administration of noradrenaline as a catecholamine was maintained. We deliberately eschewed the use of beta-agonists such as dobutamine, as these pharmaceutical agents have the potential to exacerbate the already prevalent hypercontractility at the cardiac base. We also determined that nitrite-based medications could potentially exacerbate the condition by reducing preload and afterload. Therefore, we chose not to utilize these medications. There was a gradual stabilization of blood pressure; however, the periphery remained cold. Blood pressure remained at a low level of approximately 80-90 mmHg, prompting a delay in the administration of beta-blockers upon readmission to the ICU. Additionally, beta-blockers were not the preferred treatment for cases of coronary spasm. In the nocturnal hours, instances of supraventricular tachycardia manifested, precipitating a precipitous decline in blood pressure. In response, we initiated a continuous intravenous infusion of the calcium channel blocker (Herbesser) via the CV line. The objective of this pharmaceutical intervention was twofold: firstly, to mitigate tachycardia episodes, and secondly, to forestall the onset of coronary spasm, given the uncertainty surrounding the possibility of angina arising from coronary spasm. During this period, the pacemaker rate was set to 90 beats per minute with the objective of achieving complete dependence on the pacemaker to the greatest extent possible. Subsequent to the establishment of a suitable pacing regimen, a notable stabilization in blood pressure was observed. In order to avoid reducing preload, it was imperative to maintain fluid loading and to administer diuretics sparingly and cautiously.

On 20/08/2025, we determined that early permanent pacemaker implantation was preferable. This decision was based on the consideration that if the patient had been a TC, normalization of left ventricular wall motion could take at least one week, and potentially up to one month or longer. Furthermore, we believed that while external temporary pacing fundamentally cannot achieve atrioventricular synchronization, an implantable DDD pacemaker capable of atrioventricular synchronization would be superior for optimizing cardiac function.

Another reason was a history of paroxysmal atrial fibrillation with severe bradycardia. On the same day, ventricular and atrial leads were inserted via the left axillary vein and implanted subcutaneously, connected to a dual-chamber pacemaker (Figure 4b).

**Figure 8:**
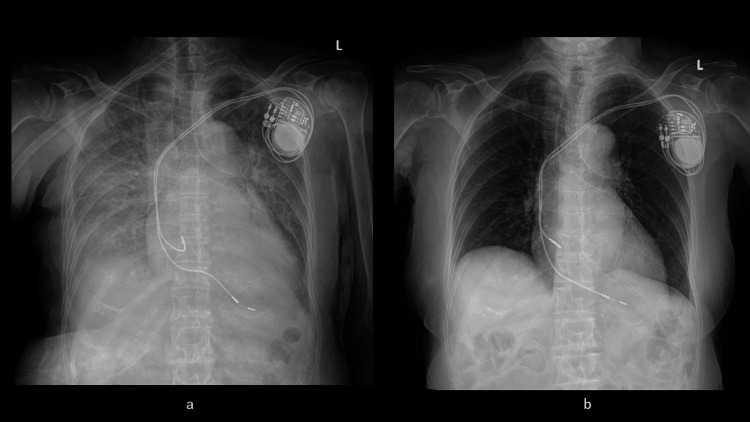
Chest X-ray films exhibiting pulmonary congestion and recovery a: Significant pulmonary congestion was observed due to substantial preload and mitral regurgitation caused by systolic anterior movement of the anterior leaflet. b: Pulmonary congestion improved with careful administration of diuretics and use of beta-blockers.

Subsequently, the external pacing lead was removed. Subsequent to this intervention, there was a substantial stabilization of hemodynamics. EC showed decreased PG due to LVOTO with SAM, leading to mitral regurgitation (Video 3). The patient's blood pressure was stabilized to around 100/55 mmHg, prompting the initiation of a continuous infusion of a low dose of beta-blocker (landiolol). The patient's intravenous fluid administration was augmented to ensure adequate preload. The patient exhibited signs of heart failure, accompanied by substantial pulmonary congestion, as evident on CXR, attributable to mitral regurgitation resulting from SAM (Figure 8a). However, oxygenation levels remained stable with the application of continuous positive airway pressure. The administration of diuretics, such as Lasix, occurred in minimal, regulated doses to circumvent a precipitous decline in preload. Consequently, pulmonary congestion accompanied by cardiac enlargement underwent a progressive resolution on CXR (Figure 8b).

**Video 3:** EC after permanent RV pacing installation Echocardiography (EC) on the day after permanent right ventricular (RV) pacing placement. Left ventricle outflow tract obstruction improved compared to the time of cardiogenic shock. Hyper systolic contractility at the left ventricular base decreased, and ballooning from the mid-ventricle to the apex also improved. Mitral regurgitation was still present.

**Figure 9:**
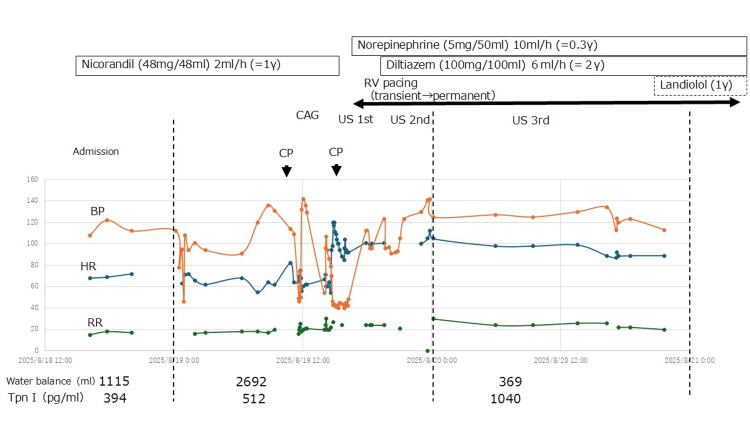
Timeline indicating clinical course of the case Cardiogenic shock due to takotsubo cardiomyopathy with left ventricle outflow tract obstruction was successfully managed with right ventricular (RV) pacing placement and beta-blocker therapy. CAG: coronary angiography, US: ultrasonography, CP: chest pain, BP: blood pressure, HR: heart rate, RR: respiratory rate, Tpn I: troponin I

While monitoring blood gas results, electrolyte imbalances were corrected, and fluids were administered to ensure adequate preload without adversely affecting oxygenation. These measures gradually elevated blood pressure, and the extremities gradually warmed (Figure 9). The patient was also delirious and required sedation with Precedex.

On 26/08/2025, beta-blockers and calcium channel blockers were switched from intravenous to oral administration.

An EC was performed on 08/09/2025, the 21st day after admission. EF had recovered to 60%, and LVOTO had decreased to 7 mmHg. At this time, the AV delay of the pacemaker was varied to observe changes in PG in the LVOT. The AV delay was set to 150 msec at this time. When the AV delay was 220 msec, PG reached its minimum value of 5 mmHg.

On 05/09/2025, an acetylcholine stress test was performed during cardiac catheterization. Oral calcium channel blockers were discontinued beforehand. No coronary artery spasm was observed, and the test was negative (Video 4).

**Video 4:** The acetylcholine stress test was negative, and there was no vasospasm of the left coronary artery

Left ventricular angiography during pacing revealed no findings of excessive contraction at the base or ballooning at the apex (Video 5).

**Video 5:** LVG after pacemaker implantation No evidence of octopus-pot-like left ventricular ballooning was observed on left ventriculography (LVG).

**Figure 10:**
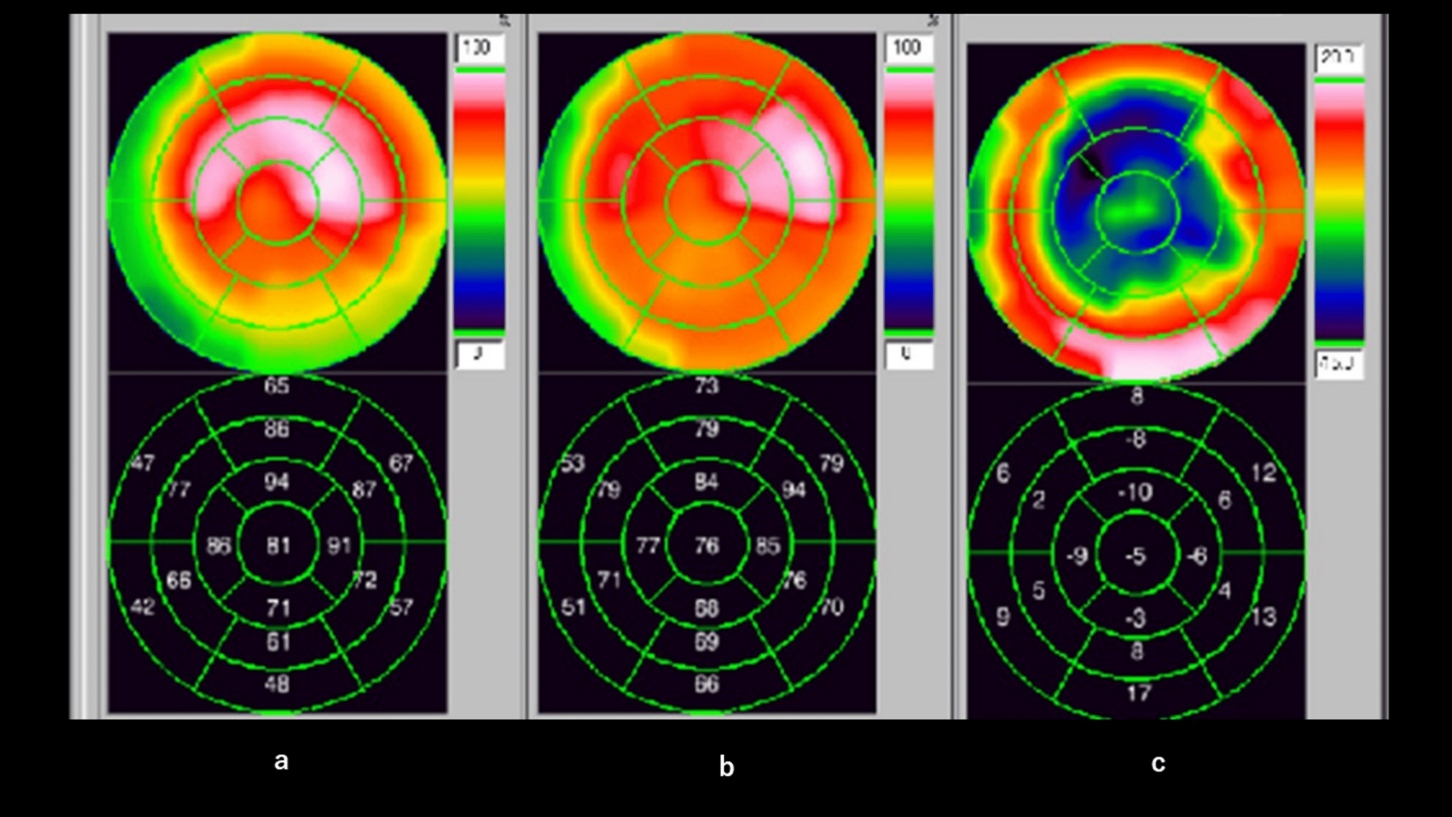
Cardiac scintigraphy with Tl-BMIPP Myocardial scintigraphy did not show a pattern of reduced blood flow along the coronary artery territory, as seen with vasospastic angina. a: thallium (Tl) scintigraphy, b: iodine-123 β-methyl-p-iodophenyl-pentadecanoic acid (BMIPP) scintigraphy, c: images of a and b

Myocardial scintigraphy was also performed, but no pattern of reduced blood flow along the coronary artery territory, as seen in vasospastic angina, was detected (Figure 10).

Based on the above, this case was diagnosed as TC with LVOTO.

**Figure 11:**
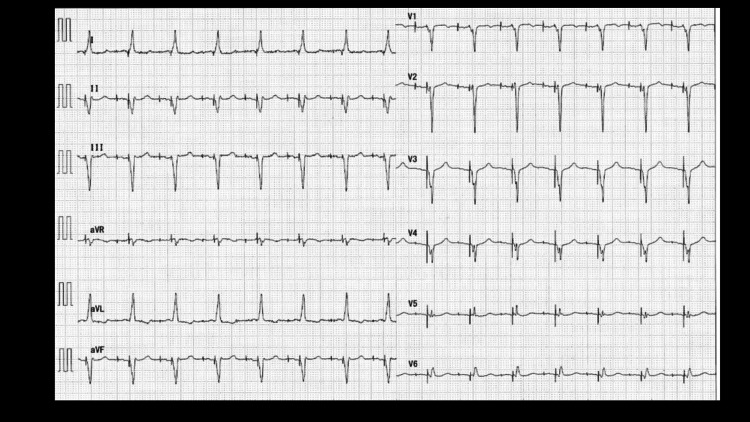
ECG at the time of discharge The ECG immediately prior to discharge showed all pacing (A pacing, V pacing) at HR 90 bpm. ECG: electrocardiogram, HR: heart rate, bpm: beats per minute

Subsequently, cardiac rehabilitation was performed, and the condition stabilized. The ECG immediately prior to discharge showed all pacing (A pacing, V pacing) at HR 90 bpm (Figure 11).

EC revealed improved left ventricular wall motion, left ventricular EF of 60%, and improved LVOT PG of 12 mmHg (Video 6).

**Video 6:** EC at the time of discharge Echocardiogram (EC) revealed improved left ventricular wall motion, left ventricular ejection fraction of 60%, and decreased left ventricular outflow tract pressure gradient to 12 mmHg.

The patient was discharged in good condition on 28/09/2025.

## Discussion

TC was first reported by Japanese physicians, including Dr. Mitsuru Sato [8]. TC is often triggered by acute emotional stress and causes transient left ventricular dysfunction with basal hyperkinesis and septal-apex ballooning, but it is not associated with significant coronary artery disease. It is said to be more common in postmenopausal women. Furthermore, it has been reported that physical stressors such as surgery or myocardial infarction can trigger takotsubo-like LVOTO, worsening hemodynamics [9]. Previously, the prognosis was considered favorable. However, currently, the in-hospital mortality rate has been reported to be approximately 4.1%, comparable to that of acute coronary syndrome, and the long-term prognosis is not favorable [2]. Circulatory instability significantly impacts mortality, particularly during the acute phase. The in-hospital mortality rate for TC complicated by cardiogenic shock reaches 28.6% [2]. Such cases involving LVOTO are reported to constitute 9.9% of all cases, and both conditions may adversely affect each other [1]. Furthermore, in TC with LVOTO complicated by cardiogenic shock, both LVOTO and SAM are thought to worsen shock progression, complicate treatment, and further deteriorate prognosis.

This patient was admitted with suspected unstable angina. Although CAG revealed normal coronary arteries, he developed cardiogenic shock with a blood pressure of 60 mmHg in the ICU. Bedside EC revealed excessive contraction at the cardiac base and bulging from the anterior-medial wall toward the apex, characteristic of TC, with a low ejection fraction of 30%. Furthermore, extremely severe LVOTO was observed, with the PG exceeding 160 mmHg due to the projection of the ventricular septum into the outflow tract and SAM of the anterior mitral leaflet. To overcome this difficult and complex situation, rapid fluid administration and norepinephrine infusion were initiated, but blood pressure did not rise sufficiently. Finally, a temporary external pacing lead was inserted into the right ventricle via the right jugular vein. Blood pressure increased upon initiation of external pacing. Bedside EC revealed a dramatic improvement in the LVOTO.

RV pacing has been used for many years to treat LVOTO in HCM, and its efficacy has been reported [3]. However, long-term improvement in prognosis has not been demonstrated, and the efficacy of RV pacing for LVOTO in HCM remains controversial. Guidelines also classify RV pacing for LVOTO in HCM as a Class IIb recommendation. To our knowledge, this report is the first to describe the efficacy of RV pacing for LVOTO-associated shock in HCM. RV pacing desynchronizes the left ventricular contraction pattern. In fact, it frequently reverses the polarity of the chest leads on the ECG and widens the QRS complex. This may result in left ventricular desynchrony, potentially leading to relief of LVOTO.

In TC, the key treatment strategies are to increase preload by administering fluids and to increase afterload by using alpha-agonists such as norepinephrine or propranolol. Although beta-blockers are considered effective, they cannot be used in shock states. Furthermore, beta-agonists such as dobutamine are contraindicated as they exacerbate excessive basal myocardial contraction [4].

In this case, RV pacing dramatically improved the LVOT PG from 160 mmHg to 107 mmHg. Furthermore, the A-line showed a marked increase in systolic blood pressure from 60 mmHg to 98 mmHg. Essentially, RV pacing induced desynchrony in the left ventricular myocardium, alleviating excessive contraction at the cardiac base and reducing LVOTO. This strategy successfully rescued the patient from shock and saved her life.

Additionally, mechanical support options include IABPs, extracorporeal membrane oxygenation (ECMO), and Impella. However, IABP alone is not suitable for this condition because it fundamentally reduces afterload. While ECMO is certainly useful for rescuing patients in shock, it essentially removes blood from the right heart system and cannot improve the blood stasis in the left atrium caused by SAM [5]. The Impella is a circulatory assist device that reduces left ventricular pressure and discharges blood flow into the aorta, appearing most suitable for improving the pathology of LVOTO [6,7]. However, its use is limited to specific facilities. Furthermore, its insertion might be difficult in low-weight patients.

Impella was not available at our hospital. The patient was an underweight woman, so even if Impella had been available, it would have been difficult to apply it to her. Temporary RV pacing placement is feasible in most standard facilities, with its major advantage being accessibility. Furthermore, as seen in this case, it also serves as a valuable backup against bradycardia caused by beta-blocker use in patients predisposed to bradycardia.

TC often resolves spontaneously, but the recovery period varies significantly from one week to one month [1,2]. When long-term temporary external pacing is required, risks such as infection and lead perforation also arise.

In this case, a permanent pacemaker was implanted the day after the shock episode. To our knowledge, this represents the first reported case of early pacemaker implantation in a patient with cardiogenic shock due to TC complicated by LVOTO. This patient had a history of bradycardia, suggesting that pacemaker implantation would have been necessary in the near future. Early permanent pacemaker implantation enabled atrioventricular synchrony. While external pacing was RV pacing without atrial synchrony, early permanent pacemaker implantation allowed atrioventricular synchrony. This further stabilized the patient's hemodynamics. By intentionally inducing left ventricular asynchrony to reduce left ventricular obstruction while simultaneously maintaining atrial-ventricular synchrony, cardiac output was improved.

Furthermore, since TC is known to recur in a certain percentage of cases, determining optimal pacing parameters remains a future research topic. Furthermore, EC was performed to achieve optimal pacing distribution during the chronic phase. AV delay was adjusted while observing changes in PG during left ventricular pacing. The results showed that PG decreased most significantly when the AV delay setting exceeded 200 msec. Considering the balance between PG and cardiac contractility, the AV delay was set with a target value of 150 msec.

The limitations of this case report include the fact that it involves only a single case, and RV pacing may have been effective by chance. To reliably confirm its efficacy, the number of reported cases must be increased. However, cardiogenic shock with concomitant LVOTO in TC is a highly dangerous condition. Dobutamine, commonly used for hypotension, is contraindicated, and beta-blockers, the most desirable treatment, are difficult to use due to hypotension. Nitrates, commonly used for angina, cannot be administered due to their preload-reducing effect. When fluid resuscitation and alpha agonists fail to effectively restore the patient from shock, RV pacing may become a realistic measure to consider in the clinical setting.

Is RV pacing appropriate for TC with non-shock-associated LVOTO? Would it be effective? For example, it may be beneficial for patients with a history of severe bradycardia. Next, it might be worth trying in TC with heart failure and LVOTO. In such cases, turning RV pacing on and off and observing the ECG and arterial line waveform might confirm whether RV pacing improves hemodynamics. If improvement occurs with pacing ON, set the pacing rate higher. Conversely, if RV pacing worsens blood pressure or PG or shows no change, revert to spontaneous rhythm. Of course, the risks of RV pacing must also be considered.

Although TC was previously regarded as having a favorable prognosis, it can, on occasion, pose a significant threat to patients. In cases of LVOTO, it is imperative to closely monitor the patient's condition using bedside EC and to adjust the treatment strategy accordingly. Further research on RV pacing is necessary to validate its efficacy and safety. Nonetheless, preliminary findings suggest that RV pacing could offer a promising new treatment option for patients in cardiogenic shock with TC and LVOTO.

## Conclusions

In a case of cardiogenic shock with TC and LVOTO, RV pacing intentionally induced left ventricular desynchrony, reduced hypercontraction at the left ventricular base, and rapidly improved LVOTO, leading to successful recovery from cardiogenic shock. Furthermore, early implantation of a dual-chamber pacemaker combined atrioventricular synchrony with ventricular desynchrony, further stabilizing hemodynamics. To the best of our knowledge, this is the first report demonstrating the efficacy of RV pacing in TC complicated by LVOTO. In this high-risk setting, when standard therapy fails, RV pacing may serve as an easily accessible and clinically useful treatment option.
